# Effects of AI on Nursing Education: Protocol for a Systematic Review and Meta-Analysis

**DOI:** 10.2196/89455

**Published:** 2026-07-31

**Authors:** Ting Yang, Bin Chen, Huai Qin

**Affiliations:** 1 Department of Nursing, Affiliated Hospital of Nanjing University of Chinese Medicine, Nanjing, Jiangsu, China; 2 Department of Medicine, Nanjing Prison, No. 9, Ningshuang Road, Yuhuatai District, Nanjing, Jiangsu, 210029, China, 86 13809035529

**Keywords:** artificial intelligence, AI, nurses, medical education and training, meta-analysis

## Abstract

**Background:**

AI demonstrates considerable potential in nursing education. However, its specific effects on knowledge acquisition, practical skills, satisfaction, competence, and confidence remain inadequately characterized.

**Objective:**

This study aims to assess the effects of AI on nursing students’ education.

**Methods:**

We will follow the PRISMA-P (Preferred Reporting Items for Systematic Reviews and Meta-Analyses Protocols) guidelines. Systematic literature searches will be conducted across 6 electronic databases, namely, PubMed, Web of Science, Embase, CINAHL, MEDLINE (EBSCOhost), and the Cochrane Library. The inclusion criteria follow the population, intervention, comparator, outcome, and study design framework, encompassing nursing students from academic institutions and clinical internship settings. This review will examine studies comparing AI-based educational interventions with traditional teaching methodologies. The outcomes will encompass knowledge level, practical ability, satisfaction, competence, and confidence. Eligible study designs include randomized controlled trials and quasi-experimental studies. The search timeline is from the inception of each database to February 2026, with no language restrictions. Two independent reviewers will screen the studies and extract data. Any disputes will be resolved through discussion. Unresolved disputes will be decided by consulting the third author. For the risk-of-bias assessment, version 2 of the Cochrane risk-of-bias tool for randomized trials and the Risk of Bias in Nonrandomized Studies of Interventions tool will be used. Moreover, the RevMan software (version 5.4) will be used for meta-analysis.

**Results:**

Literature retrieval was finished in February 2026, and formal title and abstract screening and full-text evaluation are ongoing. Data extraction, risk-of-bias assessment, and quantitative meta-analysis are scheduled to start in January 2026, with the full review manuscript planned for submission by June 2026.

**Conclusions:**

This meta-analysis will systematically quantify the overall effects of AI-assisted teaching on nursing students’ knowledge, practical ability, satisfaction, competence, and confidence. Synthesized evidence can facilitate standardized application of AI in nursing education and direct subsequent relevant research.

## Introduction

Nursing education comprises systematic educational programs designed to prepare nursing professionals [1]. It aims to equip students with nursing theory and practical skills through systematic learning and training, thereby enhancing their professional level and abilities in the field of nursing [2]. The goal of nursing education is to cultivate nursing staff with solid professional knowledge, good professional ethics, and a high sense of responsibility to meet the social demand for nursing services [3].

Traditional nursing educational methods involve teachers providing centralized face-to-face instruction to nursing students, which includes theoretical explanations and practical demonstrations [4]. Conventional methods face limitations that compromise learning efficiency and educational quality [5]. These approaches inadequately address nursing students’ needs for clinical skill development and theoretical knowledge acquisition [6]. Therefore, educational researchers have begun to explore new intervention methods to improve the learning efficiency of nursing students [7].

AI encompasses technological systems that simulate human cognitive functions through algorithmic models and computing architectures, enabling perception, reasoning, learning, and autonomous decision-making [8]. AI-enhanced education promotes student engagement and optimizes learning outcomes [9].

In recent years, with the development of AI technologies, researchers are increasingly implementing AI in nursing education contexts, playing an important and irreplaceable role in the education of nursing students [10]. These implementations create intelligent learning environments that transcend spatiotemporal constraints using technologies such as large language models, knowledge graphs, metaverse platforms, and AI-enhanced virtual simulations, thereby enhancing learning efficiency [11]. Meanwhile, machine learning and AI algorithms can analyze and interpret students’ interactions and feedback to accurately evaluate learning outcomes [12]. In addition, as the younger generation, students have a high acceptance of new technologies, and the immersive nature of AI experiences sustains engagement and promotes self-directed learning behaviors, consequently influencing learning efficacy and clinical skill development.

Nevertheless, the efficacy of AI as an instructional tool in nursing education remains contested. While some studies report knowledge enhancement [13], others demonstrate nonsignificant improvements [14,15]. Although AI demonstrates potential in improving the learning outcomes of nursing students, adequate evidence confirming the effects of AI as an assisted learning tool for nursing students is lacking.

To our knowledge, no meta-analysis has comprehensively evaluated AI’s effects in nursing education. A previous systematic review [16] investigated the effects of AI in nursing education. However, it included cross-sectional studies, qualitative studies, mixed methods studies, Delphi studies, and other types of research with a broader scope; included any topic related to AI and nursing education without restricting intervention types; and performed only qualitative narrative synthesis rather than conducting a meta-analysis restricted to randomized controlled trials (RCTs) and quasi-experimental studies. Another systematic review [17] examined the effects of generative AI on nurses’ clinical skills. However, this study focused on the development of clinical competence in nursing education across the Miller pyramid, with an emphasis on clinical competence rather than comprehensively evaluating nurses’ abilities, including knowledge, skills, satisfaction, competence, and confidence. In addition, only narrative synthesis was conducted instead of meta-analysis. Therefore, assessing the effects of AI on nursing education is urgently necessary. In this study, we aim to systematically evaluate the effectiveness of AI as a learning tool on nursing education, particularly in terms of students’ knowledge level, practical ability, satisfaction, competence, and confidence.

## Methods

### Aim

This study aims to assess the effects of AI on nursing students’ education.

### Registration

This protocol adheres to the PRISMA-P (Preferred Reporting Items for Systematic Reviews and Meta-Analyses Protocols) guidelines [18] and has been registered in PROSPERO (registration number CRD420251170836).

### Search Strategy

Electronic searches will be performed in PubMed; Web of Science; Embase; CINAHL; MEDLINE (EBSCOhost); the Cochrane Library; and gray literature sources such as ClinicalTrials.gov, the World Health Organization International Clinical Trials Registry Platform, ProQuest Dissertations & Theses Global, and key nursing conference abstracts, alongside supplementary searches via general search engines with domain restriction operators (“site:.edu,”“site:.org,” and “site:.gov.cn”) to capture unpublished research reports, technical guidelines, and institutional materials from medical universities, nursing academic associations, and health authorities. Reference snowball searching will also be conducted by screening the reference lists of all included studies to identify additional eligible studies. When full texts or essential data are unavailable, the corresponding authors will be contacted. Finally, studies containing sufficient information to assess eligibility based on the inclusion criteria will be included.

A professional librarian will be consulted to optimize the search strategies. Database-specific thesaurus terms have been added for each database: MeSH terms (PubMed, Cochrane Library, and MEDLINE [EBSCOhost]), free-text terms (Web of Science), Emtree terms (Embase), and CINAHL headings (CINAHL). Exact phrase searching using double quotation marks will be prioritized to capture fixed terminology; adjacency operators will be further adopted in CINAHL, the Cochrane Library, Embase, and Web of Science (PubMed does not support adjacency searching). The search strategy was initially developed through preliminary PubMed searches. AI-related search terms include “artificial intelligence” OR “AI” OR “Chat GPT” OR “generative artificial intelligence” OR “machine learning” OR “deep learning” OR “large language model” OR “natural language processing” OR “artificial neural networks” OR “intelligent systems.” These terms will be combined using the Boolean “OR” operator, with syntax adaptations for each database.

Search terms related to nursing education are “education, nursing” OR “nursing education” OR “nursing teaching” OR “nursing training” OR “nursing courses” OR “nursing student*.” Similarly, the Boolean operator “OR” will be used to combine the search terms with different syntaxes adapted to each database.

The 2 search concept sets will be combined using the Boolean “AND” operator, namely, “AI” and “nursing education.” The search period spans database inception to February 2026, with no language restrictions. For non–English-language literature, we will use the Baidu Translate web page [19] (https://fanyi.baidu.com/mtpe-individual/transDoc?transType=1) to translate it into English for easy reading. The references of the included studies will be searched for additional identification. The search algorithm will be developed by an experienced librarian to ensure the comprehensiveness of the literature retrieval and processing. The detailed retrieval strategy is shown in Multimedia Appendix 1.

### Eligibility Criteria

#### Population

The population includes nursing students in academic programs and nurse interns in clinical internships. “Intern” is defined as nursing students undergoing clinical practice in hospital settings.

#### Intervention

AI-facilitated learning interventions are defined as AI-supported educational activities for nursing students featuring intelligent interaction, personalized learning, adaptive feedback, automated assessment, or virtual simulation. Five distinct AI categories are predefined for inclusion: (1) large language models (eg, generative pretrained transformer series), (2) natural language processing, (3) machine learning and deep learning, (4) AI-enabled virtual simulation and intelligent training, and (5) adaptive learning systems and knowledge graphs. To mitigate heterogeneity across disparate AI modalities with divergent working mechanisms, subgroup analysis stratified by these 5 AI subgroups has been prospectively specified to quantify separate effect sizes for each technology when substantial heterogeneity emerges during meta-analysis.

Conventional instructional methods were defined as traditional face-to-face classroom teaching, lectures, and routine clinical teaching without AI support.

#### Outcome

We will assess the outcomes as follows:

- Primary outcome—knowledge level
- Secondary outcomes—practical ability, satisfaction, competence, and confidence

Knowledge level represents the measurable knowledge repository within a specific domain [20]. This construct can be assessed using standardized or custom-developed scales [21]. Practical ability denotes the systematized operational competencies developed through training [22]. It is evaluated through practical skill demonstrations [23]. Satisfaction constitutes a psychological state reflecting subjective appraisal of educational experience [24]. It can be assessed using general or self-made scales [25]. For satisfaction assessed via inconsistent rating scales across trials, the standardized mean difference (SMD) will be adopted for meta-analysis; subgroup stratification by validated or custom-made scales is predefined to explore measurement heterogeneity. Competence encompasses an individual’s capacity to appropriately execute professional tasks [26]. It can be assessed using various general instruments, such as the Nurse Competence Scale [27]. Confidence represents a psychological characteristic manifesting as self-assurance in one’s capacity to successfully perform activities, reflecting self-worth, self-respect, and self-awareness, as well as a psychological state [28]. It can be assessed using several general scales, such as the Student Satisfaction and Self-Confidence in Learning Scale [29].

#### Study Design

Eligible designs include RCTs and quasi-experimental studies comparing AI-based interventions with traditional learning approaches.

#### Exclusion Criteria

Exclusion criteria comprise (1) studies not involving nursing students; (2) AI tools used solely for administrative purposes unrelated to learning; (3) studies without a comparator group; (4) irrelevant outcomes or unavailable data; (5) reviews, editorials, notes, or errata; and (6) duplicate publications.

### Study Selection and Data Extraction

#### Study Selection

Search results will be imported into the EndNote X9 software (Clarivate Analytics) for management. First, duplicates will be identified and removed using the “Find Duplicates” function, comparing titles and authors. Second, irrelevant studies will be excluded through title and abstract screening. The third step is to screen the remaining articles from the first 2 steps, download the full texts, and exclude any research that does not meet the requirements by reading the full text. In step 4, for articles with unavailable full texts or essential data, the corresponding authors will be contacted. If this information is unrecoverable, such articles will be excluded with documented justification. Step 5 consists of reviewing the references of the finally included literature to find other studies that may meet the inclusion criteria. Two authors of this study (TY and BC) will independently conduct the literature screening. Disputes will be resolved through discussion, with unresolved issues decided by consulting the third author (HQ). The selection process follows the PRISMA (Preferred Reporting Items for Systematic Reviews and Meta-Analyses) flowchart.

#### Data Extraction

Data extraction will include the following information: (1) basic information on each study, including author, publication year, and country (or region); (2) participant characteristics, including total sample size, grouping, sample size per group, average age, gender, and students in academic programs or clinical internships; (3) characteristics of the intervention methods, including research design, specific intervention methods, duration of AI intervention, and control participants; (4) research results, including measurement methods, data types, statistical data, and outcomes (continuous outcome data will be presented as means and SDs; for data presented in alternative formats, conversion to mean and SD will follow the *Cochrane Handbook for Systematic Reviews of Interventions* guidelines); and (5) other information, including funding agency support, potential conflicts of interest, etc. Two authors (TY and BC) will independently perform data extraction. Any disputes will be resolved through discussion. Unresolved disputes should be decided by consulting with the third author (HQ). Data will be extracted using customized Microsoft Excel forms.

#### Quality Assessment of the Included Studies

For RCTs, we will use version 2 of the Cochrane risk-of-bias tool for randomized trials (ROB 2) [30]. This tool evaluates 7 domains: random sequence generation, allocation concealment, blinding of participants and personnel, blinding of outcome assessment, incomplete outcome data, selective outcome reporting, and other biases. The risk of bias level is categorized as high, unclear, or low. Nonrandomized studies will be evaluated using the Risk of Bias in Nonrandomized Studies of Interventions (ROBINS-I) tool [31]. The risk of bias includes issues related to confounding, participant selection, intervention classification, deviations from the intended interventions, missing data, outcome measurement, selection of reported results, and overall bias.

Evidence quality will be evaluated by applying the Grading of Recommendations Assessment, Development, and Evaluation (GRADE) [32] method and calculating the coefficient of agreement between raters. The κ coefficient will be classified according to the research by Landis and Koch [33] (0.0‐0.20=slight consistency, 0.21‐0.40=fair consistency, 0.41‐0.60=moderate consistency, 0.61‐0.80=substantial consistency, and 0.81‐1.00=almost perfect consistency [34]).

Two authors (TY and BC) will independently evaluate the risk of bias and quality of evidence for each included study. Any disputes will be resolved through discussion. Unresolved disputes will be decided by consulting with the third author (HQ).

### Data Synthesis and Statistical Analysis

#### Data Synthesis

Statistical analysis will be conducted using the RevMan software (version 5.4; The Cochrane Collaboration). For continuous data, the weighted mean difference (WMD) will be used for identical measurement tools; the SMD model will be applied for divergent methods. Dichotomous outcomes will be analyzed using odds ratios. All effect estimates will be reported with 95% CIs. A *P* value below .05 will indicate that the difference is statistically significant.

#### Heterogeneity Assessment

The *I*^2^ test will be used to evaluate heterogeneity levels. According to the *Cochrane Handbook for Systematic Reviews of Interventions*, there is significant heterogeneity when *I*^2^ is above 50%. If *P* is above .10 and *I*^2^ is below 50%, a fixed-effects model will be used. Otherwise, if *P* is below .10 and *I*^2^ is above 50%, a random-effects model will be applied. If conditions permit, we will collect quantitative data for meta-analysis; otherwise, the data will be presented in narrative form. Sensitivity and subgroup analyses will be conducted to explain possible sources of heterogeneity.

To address potential heterogeneity caused by different measurement tools across the included studies, we will apply the SMD to pool effect sizes for the same outcome domain assessed using different scales or instruments. For outcomes measured using identical tools and units, the WMD will be used instead. Subgroup analyses and sensitivity analyses will be performed to explore and explain sources of heterogeneity. If significant heterogeneity exists (*I*^2^>50%), a random-effects model will be used for meta-analysis.

#### Subgroup Analysis

If significant heterogeneity (*I*^2^>50%) is found and the source of heterogeneity cannot be detected through sensitivity analysis, subgroup analysis will be performed. Subgroup analysis may examine type of AI technology (large language models, virtual simulation, adaptive learning systems, and other AI technologies), intervention modality (theoretical teaching, skill training, and integrated teaching), measurement tool, study design, study characteristics, participant demographics, intervention type, intervention duration, sample size, and other aspects.

#### Sensitivity Analysis

When heterogeneity is significant, the leave-one-out method will be conducted to determine whether it is caused by a particular study. For example, we will remove one study to determine whether heterogeneity is reduced. This method will be used to test each study to identify potential sources of heterogeneity.

#### Publication Bias Assessment

For meta-analysis involving 10 or more studies, we will use funnel plots to assess publication bias levels. Specifically, this method evaluates the symmetry of the funnel plot through visual inspection and the Egger test, with a significance level of 5% [35]. For analyses with fewer than 10 studies, publication bias will be qualitatively assessed based on study characteristics.

#### Evidence Quality

The GRADE approach will be used to assess the quality of each piece of evidence [36]. Evidence quality will be rated as high, moderate, low, or very low based on the risk of bias, inconsistency, indirectness, imprecision, and publication bias [37]. Initial high-quality ratings may be downgraded according to deficiencies in each domain. Evidence quality may be upgraded when factors strongly enhance confidence in effect estimates. Two authors (TY and BC) will independently rate each comparison area, resolving differences through consensus. Unresolved disputes will be decided by consulting the third author (HQ).

### Patient and Public Involvement

Our research does not involve patients or the public in the design, execution, or planning of reporting and dissemination.

With the continuous improvement and development of AI technology, it has been applied in clinical nursing education research and achieved satisfactory results [38]. Previous studies have shown that AI plays a role in assisting nursing students’ learning, but the specific effects are still controversial [13-15]. We will further analyze which aspects of AI have a positive impact on the learning of nursing students, which aspects have no effect, and which aspects have adverse effects. We will also explore possible reasons for this. How to maximize the advantages of AI in nursing education will become a future development direction. This concept provides a more scientific intervention plan and theoretical basis for the application of AI in nursing education, which has clinical significance.

### Ethical Considerations

This study does not involve clinical research and, thus, does not require ethics approval.

## Results

This protocol was registered with PROSPERO (CRD420251170836) in October 2025. The systematic literature search was conducted after protocol registration in February 2026, whereas formal title and abstract screening and full-text evaluation are ongoing. Data extraction, risk-of-bias assessment, and quantitative meta-analysis are scheduled to start in January 2026, with the full review manuscript planned for submission by June 2026.

## Discussion

### Principal Findings

This meta-analysis is expected to quantify pooled effect sizes of AI-assisted teaching vs conventional nursing education across 5 outcomes: knowledge level, practical ability, satisfaction, competence, and confidence. We hypothesize that AI will produce moderate overall improvements in primary knowledge level, whereas effect magnitudes may vary substantially for practical ability, satisfaction, competence, and confidence owing to diversified AI modalities and evaluation instruments. Subgroup analyses are anticipated to reveal divergent efficacy among large language models, virtual simulation, and adaptive learning systems.

### Comparison With Existing Literature

Available prior systematic reviews on AI in nursing education have mostly adopted a qualitative synthesis approach and included uncontrolled cross-sectional or mixed methods research without being restricted to RCT and quasi-experimental designs. One earlier review [17] focused exclusively on generative AI effects mapped to the Miller clinical competence framework rather than comprehensively synthesizing 5 multidimensional learning indicators. Our planned quantitative meta-analysis fills this gap by limiting eligible study types to comparative controlled trials and performing pooled effect calculation, enabling more robust evidence synthesis unavailable from previous narrative reviews.

### Strengths and Limitations

#### Strengths

First, strict adherence to PRISMA-P guidelines and prospective PROSPERO registration reduce publication bias risk and enhance research transparency. Second, dual independent literature screening and risk-of-bias appraisal with third-party arbitration reduce selection and assessment bias, and the combined use of the ROB 2 and ROBINS-I tools and the GRADE framework ensures rigorous methodological and evidence grading. Third, prespecified subgroup and sensitivity analyses are set to systematically explore sources of between-study heterogeneity.

#### Limitations

Heterogeneity is highly probable due to inconsistent AI intervention types, variable intervention durations, and disparate measurement scales across included trials, which may restrict the robustness of the pooled results. In addition, unpublished gray literature cannot be fully retrieved, potentially introducing small publication bias.

### Future Research Directions and Dissemination Plan

Future primary trials should standardize AI intervention specifications and unify outcome measuring tools to reduce between-study inconsistency. Subsequent research can further develop stratified AI teaching schemes tailored to nursing students vs clinical interns. For dissemination, finalized meta-analysis results will be submitted to peer-reviewed academic journals focused on nursing education or digital health; core findings will also be summarized for local nursing teaching conferences and institutional nursing curriculum revision meetings to facilitate real-world translation of AI education evidence into routine teaching practice.

### Conclusions

Once completed, this meta-analysis will generate quantitative evidence clarifying the overall benefits of AI-assisted nursing education against traditional instruction, resolve inconsistent conclusions from existing narrative reviews, and deliver evidence-based references for standardized AI integration into nursing curriculum design.

## Supplementary material

10.2196/89455 Multimedia Appendix 1 Detailed Retrieval Strategy

## Abbreviations

GRADE: Grading of Recommendations Assessment, Development, and Evaluation
PRISMA: Preferred Reporting Items for Systematic Reviews and Meta-Analyses
PRISMA-P: Preferred Reporting Items for Systematic Reviews and Meta-Analyses Protocols
RCT: randomized controlled trial
ROB 2: version 2 of the Cochrane risk-of-bias tool for randomized trials
ROBINS-I: Risk of Bias in Nonrandomized Studies of Interventions
SMD: standardized mean difference
WMD: weighted mean difference
