# Supplementary material for: Effects of AI on Nursing Education: Protocol for a Systematic Review and Meta-Analysis
Source: JMIR Res Protoc. 2026 Jul 31;15:e89455. doi: 10.2196/89455 (PMC13433342; doi:10.2196/89455)
Supplement: Supplementary file 1 [file resprot-v15-e89455-s001.docx]

## Appendix:

## Search strategies

PubMed

#1. Artificial Intelligence [MeSH Terms]

#2. Generative Artificial Intelligence [MeSH Terms]

#3. Machine Learning [MeSH Terms]

#4. Deep Learning [MeSH Terms]

#5. Large Language Models [MeSH Terms]

#6. Natural Language Processing [MeSH Terms]

#7. Intelligent Systems [MeSH Terms]

#8. ((((((((((artificial intelligence [Title/Abstract])) OR (AI [Title/Abstract])) OR (Chat GPT [Title/Abstract])) OR (generative artificial intelligence [Title/Abstract])) OR (machine learning [Title/Abstract])) OR (deep learning [Title/Abstract])) OR (large language model [Title/Abstract])) OR (natural language processing [Title/Abstract])) OR (artificial neural networks [Title/Abstract])) OR (intelligent system [Title/Abstract]))

#9. #1 OR #2 OR #3 OR #4 OR #5 OR #6 OR #7 OR #8

#10. Students, Nursing [MeSH Terms]

#11. Nursing Staff [MeSH Terms]

#12. ((((((education, nursing [Title/Abstract])) OR (nursing education [Title/Abstract])) OR (nursing teaching [Title/Abstract])) OR (nursing training [Title/Abstract])) OR (nursing courses [Title/Abstract])) OR (nursing student* [Title/Abstract]))

#13. #10 OR #11 OR #12

#14. #9 AND #13

Web of Science

#1. ‎(‎(‎(‎(‎(‎(‎(‎(‎((‎Subject: (artificial intelligence) OR Subject: (‎AI) OR Subject: (Chat GPT) OR Subject: (generative artificial intelligence) OR Subject: (machine learning) OR Subject: (deep learning) OR Subject: (‎large language model ) OR Subject: (‎natural language processing) OR Subject: (‎artificial neural networks) OR Subject: (‎intelligent systems)

#2. ((((((education, nursing) OR Subject: (nursing education) OR Subject: (nursing teaching) OR Subject: (nursing training) OR Subject: (nursing courses) OR Subject: (nursing student*)

#3. #1 AND #2

EMBASE

#1. “artificial intelligence”/exp OR “machine learning”/exp OR “deep learning”/exp OR “natural language processing”/exp OR “neural network”/exp

#2. (artificial intelligence):ti,ab,kw OR (AI):ti,ab,kw OR (Chat GPT):ti,ab,kw OR (generative artificial intelligence):ti,ab,kw OR (machine learning):ti,ab,kw OR (deep learning):ti,ab,kw OR (large language model):ti,ab,kw OR (natural language processing):ti,ab,kw OR (artificial neural networks):ti,ab,kw OR (intelligent systems):ti,ab,kw

#3. #1 OR #2

#4. “nursing student”/exp OR “nursing education”/exp

#5. (education, nursing):ti,ab,kw OR (nursing education):ti,ab,kw OR (nursing teaching):ti,ab,kw OR (nursing training):ti,ab,kw OR (nursing courses):ti,ab,kw OR (nursing student*):ti,ab,kw

#6. #4 OR #5

#7. #3 AND #6

CINAHL

#1. MH (Artificial Intelligence) OR MH (Machine Learning) OR MH (Natural Language Processing) OR MH (Neural Networks)

#2. ‎TX (artificial intelligence) OR TX (AI) OR TX (‎Chat GPT) OR TX (generative artificial intelligence) OR TX (machine learning) OR TX (deep learning) OR TX (large language model) OR TX (natural language processing) OR TX (artificial neural networks) OR TX (intelligent systems)

#3. #1 OR #2

#4. MH (Nursing Students) OR MH (Nursing Education)

#5. TX (education, nursing) OR TX (nursing education) OR TX (nursing teaching) OR TX (nursing training) OR TX (nursing courses) OR TX (nursing student*)

#6. #4 OR #5

#7. #3 AND #6

MEDLINE (EBSCOhost)

#1. Artificial Intelligence [MeSH Terms]

#2. Generative Artificial Intelligence [MeSH Terms]

#3. Machine Learning [MeSH Terms]

#4. Deep Learning [MeSH Terms]

#5. Large Language Models [MeSH Terms]

#6. Natural Language Processing [MeSH Terms]

#7. Intelligent Systems [MeSH Terms]

#8. ((((((((((artificial intelligence [Title/Abstract])) OR (AI [Title/Abstract])) OR (Chat GPT [Title/Abstract])) OR (generative artificial intelligence [Title/Abstract])) OR (machine learning [Title/Abstract])) OR (deep learning [Title/Abstract])) OR (large language model [Title/Abstract])) OR (natural language processing [Title/Abstract])) OR (artificial neural networks [Title/Abstract])) OR (intelligent system [Title/Abstract]))

#9. #1 OR #2 OR #3 OR #4 OR #5 OR #6 OR #7 OR #8

#10. Students, Nursing [MeSH Terms]

#11. Nursing Staff [MeSH Terms]

#12. ((((((education, nursing [Title/Abstract])) OR (nursing education [Title/Abstract])) OR (nursing teaching [Title/Abstract])) OR (nursing training [Title/Abstract])) OR (nursing courses [Title/Abstract])) OR (nursing student* [Title/Abstract]))

#13. #10 OR #11 OR #12

#14. #9 AND #13

the Cochrane Library

#1. Artificial Intelligence [MeSH Terms]

#2. Generative Artificial Intelligence [MeSH Terms]

#3. Machine Learning [MeSH Terms]

#4. Deep Learning [MeSH Terms]

#5. Large Language Models [MeSH Terms]

#6. Natural Language Processing [MeSH Terms]

#7. Intelligent Systems [MeSH Terms]

#8. (artificial intelligence):ti,ab,kw OR (AI):ti,ab,kw OR (Chat GPT):ti,ab,kw OR (generative artificial intelligence):ti,ab,kw OR (machine learning):ti,ab,kw OR (deep learning):ti,ab,kw OR (large language model):ti,ab,kw OR (natural language processing):ti,ab,kw OR (artificial neural networks):ti,ab,kw OR (‎intelligent systems):ti,ab,kw

#9. #1 OR #2 OR #3 OR #4 OR #5 OR #6 OR #7 OR #8

#10. Students, Nursing [MeSH Terms]

#11. Nursing Staff [MeSH Terms]

#12. (education, nursing):ti,ab,kw OR (nursing education):ti,ab,kw OR (nursing teaching):ti,ab,kw OR (nursing training):ti,ab,kw OR (nursing courses):ti,ab,kw OR (nursing student*):ti,ab,kw

#13. #10 OR #11 OR #12

#14. #9 AND #13

Grey Literature

(1) ClinicalTrials.gov

#1.‎ (“artificial intelligence” OR “AI” OR “‎Chat GPT” OR “generative artificial intelligence” OR “machine learning” OR “deep learning” OR “large language model” OR “natural language processing” OR “artificial neural networks” OR “intelligent systems”)

#2. (“education, nursing” OR “nursing education” OR “nursing teaching” OR “nursing training” OR “nursing courses” OR “nursing student*”)

#3. #1 AND #2

(2) WHO ICTRP

#1.‎ (“artificial intelligence” OR “AI” OR “‎Chat GPT” OR “generative artificial intelligence” OR “machine learning” OR “deep learning” OR “large language model” OR “natural language processing” OR “artificial neural networks” OR “intelligent systems”)

#2. (“education, nursing” OR “nursing education” OR “nursing teaching” OR “nursing training” OR “nursing courses” OR “nursing student*”)

#3. #1 AND #2

(3) ProQuest Dissertations & Theses Global

#1.‎ (“artificial intelligence” OR “AI” OR “‎Chat GPT” OR “generative artificial intelligence” OR “machine learning” OR “deep learning” OR “large language model” OR “natural language processing” OR “artificial neural networks” OR “intelligent systems”)

#2. (“education, nursing” OR “nursing education” OR “nursing teaching” OR “nursing training” OR “nursing courses” OR “nursing student*”)

#3. #1 AND #2

(4) Conference abstract manual search

#1.‎ (“artificial intelligence” OR “AI” OR “‎Chat GPT” OR “generative artificial intelligence” OR “machine learning” OR “deep learning” OR “large language model” OR “natural language processing” OR “artificial neural networks” OR “intelligent systems”)

#2. (“education, nursing” OR “nursing education” OR “nursing teaching” OR “nursing training” OR “nursing courses” OR “nursing student*”)

#3. #1 AND #2

(5) Supplementary retrieval

#1. (“artificial intelligence” OR “AI” OR “‎Chat GPT” OR “generative artificial intelligence” OR “machine learning” OR “deep learning” OR “large language model” OR “natural language processing” OR “artificial neural networks” OR “intelligent systems”) AND (“education, nursing” OR “nursing education” OR “nursing teaching” OR “nursing training” OR “nursing courses” OR “nursing student*”) file type: pdf

#2. Backward snowball: review reference lists of all included full-text articles for unpublished grey papers.
